# The Association Between Chronic Pain, Substance use, and Primary Care Experience Among Veterans with Ongoing or Recent Homelessness

**DOI:** 10.1007/s11606-024-09078-x

**Published:** 2024-10-15

**Authors:** Allyson L. Varley, Aerin J. DeRussy, Audrey L. Jones, April Hoge, Adam J. Gordon, Joshua Richman, Kevin R. Riggs, Lillian Gelberg, Sonya Gabrielian, John R. Blosnich, Ann Elizabeth Montgomery, Evan Carey, Stefan G. Kertesz

**Affiliations:** 1 Birmingham VA Health Care System, 700 19th Street South, Birmingham, AL 35233 USA; 2 UAB Heersink School of Medicine, Birmingham, USA; 3 VA Salt Lake City Health Care System, Salt Lake City, USA; 4 University of Utah School of Medicine, Salt Lake City, USA; 5 UCLA David Geffen School of Medicine, Los Angeles, USA; 6 VA Greater Los Angeles Health Care System, Los Angeles, USA; 7 University of Southern California, Los Angeles, USA; 8 Center for Health Equity Research and Promotion, VA Pittsburgh Healthcare System, Pittsburgh, USA; 9 UAB School of Public Health Birmingham, Birmingham, USA; 10 University of Colorado School of Public Health, Aurora, USA; 11 Rocky Mountain Regional VA Medical Center, Aurora, USA

**Keywords:** chronic pain, primary care experience, survey research, homelessness, substance use

## Abstract

**Background:**

Chronic pain and problematic substance use are prevalent among Veterans with homeless experience (VHE) and may contribute to a challenging primary care experience.

**Objective:**

To examine the association of chronic pain and problematic substance use with unfavorable primary care experiences among VHE and to explore the association of pain treatment utilization and unfavorable care experiences in VHE with chronic pain.

**Methods:**

We surveyed VHE (*n* = 3039) engaged in homeless-tailored primary care at 29 Veterans Affairs Medical Centers (VAMCs). We assessed unfavorable primary care experiences with four validated *Primary Care Quality-Homeless* (PCQ-H) scales: multivariable logistic regressions explored associations between unfavorable care experiences for VHE with chronic pain and problematic substance use, chronic pain alone, problematic substance use alone, or neither. We then examined the association between receipt of pain treatments and unfavorable experiences among VHE with chronic pain. Last, we identified PCQ-H items that had the greatest difference in unfavorable response rates between VHE with and without chronic pain.

**Results:**

The prevalence of unfavorable primary care experience was higher on all four scales for patients reporting chronic pain (with or without problematic substance use) (all *p* < 0.001), but not for problematic substance use alone, compared to VHE with neither pain nor problematic substance use. In analyses limited to VHE with chronic pain, those on long-term opioids were less likely to report an unfavorable experience (OR = 0.49, 95%CI 0.34–0.69). Receipt of occupational therapy was associated with lower odds of reporting an unfavorable experience (OR = 0.83, 95%CI 0707–0.98). PCQ-H items related to trust, relationships, and provider communication had the greatest differences in dissatisfaction ratings (all *p* < 0.001).

**Conclusions:**

Chronic pain is associated with unfavorable primary care experiences among VHE, potentially contributing to poor care outcomes. Strategies are needed to enhance patient-provider trust and communication and increase VHE’s access to effective pain treatments.

**Supplementary Information:**

The online version contains supplementary material available at 10.1007/s11606-024-09078-x.

## INTRODUCTION

An estimated 582,000 Americans, including 33,129 Veterans, were homeless on a single night in 2022,^1^ and more were homeless over the course of a year.^2^ Physical and mental illness represent major clinical concerns and contribute to mortality.^3–5^ Chronic pain and substance use disorders (SUD) are prevalent ^6–9^ and contribute to disability for Veterans with homeless experience (VHE). Pain may precede other symptoms^10^ and concerns regarding potential problematic substance use may complicate discussion of medication decisions for pain management.^8,11^ Experts recommend an integrative multidimensional pharmaco-behavioral approach to pain treatment.^12,13^ However, for persons with homeless experience, integrative approaches may be difficult to obtain^14–16^ due in part to poverty and unstable housing, which present competing priorities,^14^ including access to food and shelter, transportation, lack of storage, and weak trust in health systems.

Primary care clinics are positioned to deliver the integrated care needed for persons experiencing homelessness, pain, and/or SUD. When credible patient-provider relationships and timely access to care are available, the best healthcare may mitigate the impact of pain, address risks of medical conditions, and manage treatments. This ideal may be challenged by a lack of “mutual misunderstanding” regarding pain between patients and clinicians.^17–21^ In a study of perceptions of care among individuals experiencing homelessness, 27 interviewees — clinicians and patients — raised unprompted concerns about pain including opioid medication as emblematic of problematic overlap among pain, substance use, and stigmatization.^18^

Quantifying the role of chronic pain and problematic substance use in primary care experience could help health systems improve care for VHE. Valid ratings of care experience are not interchangeable with “satisfaction” (i.e., the degree to which the patient was pleased). Rather, validated surveys of experience capture the interplay of expectations, the care-seeking process, and services received.^22^ Quantitative evaluation of patients’ care experience could help health systems understand what they are accomplishing for VHE.

Using data from a national survey of VHE, we sought to examine the associations of both self-reported chronic pain and problematic substance use to primary care experiences for VHE. We hypothesized that pain and problematic substance use would each independently be associated with worse care experiences. We also explored whether patients’ receipt of pain treatments was associated with unfavorable primary care experiences. Last, we explored which specific items on a primary care experience questionnaire were correlated with poor care experience.

## METHODS

### Overview

This study utilized primary care experience–focused survey data collected between April and October of 2018 from VHE enrolled in 29 homeless-tailored primary care clinics.^23^ Details of this survey’s collection are published elsewhere.^24^ Briefly, the survey was administered to Veterans that had at least two primary care visits in the 24 month study period and evidence of homelessness in administrative records. All Veterans were assigned to a single Homeless Patient-Aligned Care Team, a program that began in 2012.^25,26^ Study procedures were approved by VA’s Central Institutional Review Board.

### Data Sources

We linked VHE’s survey responses to their electronic health record, including sociodemographics, diagnoses, and receipt of health services. Measures of primary care experience, psychological symptoms, social support, pain, and problematic substance use were derived from survey responses.

### Measures

#### Primary Outcome

The primary outcome of interest was an overall unfavorable primary care experience as assessed by the validated *Primary Care Quality-Homeless* (PCQ-H) instrument.^27^ The survey queries perceptions of the patient’s regular source of primary care at the time the survey is administered. The PCQ-H provides four scales measuring *Relationship* to provider, perceived *Cooperation* among clinicians, *Accessibility/Coordination*, and *Homeless-specific Needs*. It relies on Likert-type items ranging 1 (worst) to 4 (best), with reverse-scoring for items where agreement indicates a worse experience. Prior studies designate an unfavorable experience on each PCQ-H scale based on the number of unfavorable item responses falling into the highest tertile for that scale.^23,28^ An unfavorable response is present when the individual disagrees with a positive PCQ-H item or agrees with a negative PCQ-H item. We assigned an overall unfavorable experience designation to Veterans whose number of unfavorable responses fell into the highest tertile of unfavorable responses on > 2 of 4 PCQ-H scales. A categorical unfavorable experience is of interest because patients have reported that responses to pain often result in poor care experiences,^18^ and because a categorical measure for such an experience is readily appreciated by clinicians. See Supplementa1 Table 1 for sensitivity analyses examining multivariable-adjusted odds ratio for unfavorable primary care experience by definition of unfavorable experience.

#### Primary Covariates

Self-reported problematic substance use was defined as affirming “Yes” to both items in the Two-Item Conjoint Screen for Alcohol and Other Drug Problems.^29,30^ The affirmation of two items on the TICS had a positive predictive value of 72% for current DSM-III-R Drug Abuse or Drug Dependence in primary care, and is likely higher among VHE. Although the current study aims at substance use disorder, a screen is not a diagnostic instrument, so we have applied “problematic substance use” language to encompass what it captures. Chronic pain was defined as reporting both (1) bodily pain that has lasted more than 3 months (modified Brief Chronic Pain Questionnaire^31,32^) and (2) average pain severity of ≥ 4 out of 10.^33^ We devised four groups: chronic pain and problematic substance use, chronic pain alone, problematic substance use alone, or neither. Sources used to measure primary covariates are described in Table 1.

**Table 1:** Sources for Chronic Pain and Substance Use Variables

Domain	Questions	Source
Self-reported problematic substance use	1. In the last **12 months**, have you ever drunk alcohol or used drugs more than you meant to? 2. Have you felt you wanted or needed to cut down on your drinking or drug use in the **last 12 months**?	Brown RL, Leonard T, Saunders LA, Papasouliotis O. A two-item conjoint screen for alcohol and other drug problems. J Am Board *Fam Pract*. Mar-Apr 2001;14(2):95–106 Brown RL, Rounds LA. Conjoint screening questionnaires for alcohol and other drug abuse: criterion validity in a primary care practice. *Wis Med J.* 1995;94(3):135–40
Self-reported chronic pain	3. Please **circle the number** that best describes your pain on average in the past week (≥ 4) 4. Do you currently have bodily pain that has lasted for **more than 3 months**?	Landmark T, Romundstad P, Dale O, Borchgrevink PC, Kaasa S. Estimating the prevalence of chronic pain: validation of recall against longitudinal reporting (the HUNT pain study). *Pain.* 2012 Jul;153(7):1368–1373 Merlin JS, Westfall AO, Chamot E, Saag M, Walcott M, Ritchie C, Kertesz S. Quantitative Evaluation of an Instrument to Identify Chronic Pain in HIV-Infected Individuals. *AIDS Res Hum Retroviruses.* 2015 Jun;31(6):623–7 Krebs EE, Lorenz KA, Bair MJ, Damush TM, Wu J, Sutherland JM, Asch SM, Kroenke K. Development and initial validation of the PEG, a three-item scale assessing pain intensity and interference. *J Gen Intern Med*. 2009 Jun;24(6):733–8

#### Pain-Related Services

We quantified opioid receipt and services related to pain management in the 12 months preceding November 2017. We defined long-term opioid therapy (LTOT) as ≥ 56 days of opioids prescribed within each quarter of the 12 months prior to survey completion.^34^ Pain management services were based on encounters for physical therapy, active therapy, occupational therapy, or pain clinic using a combination of CPT codes, ICD9/10 codes, and/or VA clinic stop codes (Supplementary Table 2).^35^

#### Other Covariates

We quantified VA service use in the 24 months prior to November 2017. The numbers of primary care and emergency department visits were designated as “high” based on actual distribution: persons in the top third of primary care use (> 5 visits in 24 months) and persons in the top 10% of emergency department use (> 8 visits in 24 months). Other covariates were based on predisposing, enabling/impeding, and need factors in the Andersen-Gelberg Behavioral Model for Vulnerable Populations,^36^ and characteristics associated with care experiences in prior studies.^23^ Predisposing characteristics included age, gender, race, and marital status. Enabling/impeding characteristics included chronic homelessness, difficulty paying for basic needs, and justice involvement. We adapted a social support scale combining four “Emotional Support” items in the National Institutes of Health Patient-Reported Outcomes Measurement Information System (PROMIS): one item from its Social Isolation scale^37^ and one for capacity to borrow $20 (Cronbach *α* = 0.84). Need characteristics included self-reported general health^38^ and serious psychological distress, based on four items from the PHQ-4^39^ and two symptoms from the Colorado Mental Health Symptom Index (Cronbach *α* = 0.84).^40,41^ Psychological distress was dichotomized at ≥ 10 to indicate “severe,” attainable by reporting five of six symptoms “several days” a week.

#### Analysis

We use cross-tabulations to compare the four groups (i.e., self-reported chronic pain and problematic substance use, chronic pain alone, problematic substance use, or neither). In multivariable logistic regression, the four groups were first treated as a categorical variable with 3 degrees of freedom. Because of a priori hypothesis that the four groups would differ, our initial analysis separated the groups. Post hoc, we tested for an interaction of pain and problematic substance use. All models included a random intercept for VA site (*n* = 26), with inverse response weights based on modeled propensity of survey response, derived from comparison of respondents to non-respondents.^23^ To assess for a potential association between pain service receipt and primary care ratings, we restricted the cohort to respondents with chronic pain, applying the same multivariable logistic regression approach and adjusting for the same covariates.

Finally, we considered which specific PCQ-H survey items obtained the most disparate responses between patients with and without chronic pain, to expose aspects of care that might require attention. In this post hoc analysis, for each of the 33 items in the PCQ-H survey, we fit a logistic regression model for unfavorable response by chronic pain adjusting for three characteristics strongly associated with unfavorable experience in prior work (self-reported general health, unsheltered status, and psychological distress)^23^. For each item, the strength of the association between chronic pain and unfavorable experience was quantified by the chi-square statistic. We then ranked the items by chi-square statistic and present those with the ten largest values.

## RESULTS

VHE (*n* = 3394) responded to the PCQ-HoST survey (37.3% response rate). Among these, 3039 had complete data on pain, problematic substance use, and the four PCQ-H rating scales. There were 355 (10%) Veterans missing data on outcomes of interest and they were excluded from the analysis. There were some statistical differences between those with and without complete information on these variables. Notably, respondents with complete data were somewhat more likely to be older, obtain education past high school or GED, and report high levels of social support; and less likely to be employed or report high primary care use (Supplemental Table 3).

### Characteristics of the Sample

Among 3039 Veterans, 12% (*n* = 352) reported problematic substance use only, 37% (*n* = 1129) chronic pain only, and 24% (*n* = 731) chronic pain and problematic substance use, while 27% (*n* = 827) reported neither. Demographic differences were observed across the four groups (Table 2). Compared to the group distribution, VHE with neither problematic substance use nor pain were disproportionately older (65 +) and retired. Those with chronic pain only were disproportionately female and married, and reported poor health and difficulty paying for basic needs. Those with chronic pain and problematic substance use disproportionately had criminal justice involvement, chronic homelessness, mental distress, and difficulty paying for basic needs.

**Table 2:** Characteristics of the Sample by Four Groups: None, Problematic Substance Use Only, Chronic Pain Only, or Both

	Overall	None	Problematic substance use only	Chronic pain only	Both	*p*-value*
	n (%)	n (%)	n (%)	n (%)	n (%)	
	3039 (100.0)	827 (27.2)	352 (11.6)	1129 (37.2)	731 (24.1)	
Race						
African American	1233 (40.57)	307 (37.12)	150 (42.61)	444 (39.33)	332 (45.42)	
Caucasian	1183 (38.93)	364 (44.01)	140 (39.77)	435 (38.53)	244 (33.38)	0.005
Hispanic	318 (10.46)	84 (10.16)	35 (9.94)	123 (10.89)	76 (10.40)	
Other	295 (9.71)	72 (8.71)	26 (7.39)	119 (10.54)	78 (10.67)	
Marital status						
Married/partnered	446 (14.68)	104 (12.58)	34 (9.66)	194 (17.18)	114 (15.60)	
Never married	855 (28.13)	270 (32.65)	125 (35.51)	275 (24.36)	185 (25.31)	< 0.001
Divorced/separated	1501 (49.39)	384 (46.43)	173 (49.15)	570 (50.49)	374 (51.16)	
Widowed	189 (6.22)	58 (7.01)	15 (4.26)	72 (6.38)	44 (6.02)	
Sex						
Female	164 (5.48)	34 (4.11)	13 (3.69)	82 (7.26)	35 (4.79)	.005
Age						
18–50	797 (26.23)	196 (23.70)	115 (32.67)	276 (24.45)	210 (28.73)	
51–64	1744 (57.39)	445 (53.81)	194 (55.11)	656 (58.10)	449 (61.42)	< 0.001
65 +	498 (16.39)	186 (22.49)	43 (12.22)	197 (17.45)	72 (9.85)	
Education						
HS/GED	1245 (42.30)	332 (40.15)	148 (42.05)	453 (40.12)	312 (42.68)	0.44
Employment status						
Employed	623 (21.00)	232 (28.05)	80 (22.73)	202 (17.89)	109 (14.91)	
Unemployed	1717 (57.89)	357 (43.17)	201 (57.10)	666 (58.99)	493 (67.44)	< 0.001
Retired	626 (21.11)	225 (27.21)	63 (17.90)	230 (20.37)	108 (14.77)	
Monthly income < $1000						
Yes	1355 (45.81)	332 (40.15)	158 (44.89)	505 (44.73)	360 (49.25)	0.004
Difficulty paying for basics						
Yes	771 (25.86)	121 (14.63)	68 (19.32)	342 (30.29)	240 (32.83)	< 0.001
Chronically homeless						
Yes	669 (22.01)	133 (16.08)	71 (20.17)	225 (19.93)	240 (32.83)	< 0.001
Criminal record						
Yes	786 (26.08)	155 (18.74)	112 (31.82)	263 (23.29)	256 (35.02)	< 0.001
Jail/prison in last year						
Yes	228 (7.52)	29 (3.51)	39 (11.08)	60 (5.31)	100 (13.68)	< 0.001
Primary care usage						
Top tertile	794 (26.13)	249 (30.11)	124 (35.23)	267 (23.65)	154 (21.07)	
Middle tertile	1066 (35.08)	294 (35.55)	129 (36.65)	380 (33.66)	263 (35.98)	< 0.001
Bottom tertile	1179 (38.80)	284 (34.34)	99 (28.13)	482 (42.69)	314 (42.95)	
Emergency room usage						
High usage (top 10%)	262 (8.6)	42 (5.08)	32 (9.09)	85 (7.53)	103 (14.09)	< 0.001
Social support						
High	1852 (61.53)	596 (72.07)	216 (61.36)	662 (58.64)	378 (51.71)	< 0.001
Mental distress						
High	1002 (33.15)	130 (15.72)	91 (25.85)	395 (34.99)	386 (52.80)	< 0.001
Self-reported health						
Poor/fair	1364 (46.47)	207 (25.03)	99 (28.13)	645 (57.13)	413 (56.50)	< 0.001
Chronic pain **						
Yes	1860 (61.20)	0 (0.00)	0 (0.00)	1129 (100%)	731 (100%)	-
SU problem in last 12 months **						
Yes	1083 (100%)	0 (0.00)	352 (32.50)	0 (0.00)	731 (67.50)	-

^*^p-values are from *x*^2^

^**^Self-reported chronic pain and self-reported problematic substance use

### Unfavorable Experience Comparison

Overall, patients with chronic pain were more likely to report unfavorable care experiences compared to VHE without chronic pain (Table 3): 39.5% of those with chronic pain only and 43.6% of those with chronic pain and problematic substance use reported unfavorable care experiences, compared to 25.3% and 22.0% of those with problematic substance use only or neither pain nor problematic substance use (*p* < 0.001). Results were consistent across the PCQ-H subscales.

**Table 3:** Negative Responses Across PCQ-H Domains by Study Group: None, Problematic Substance Use Only, Chronic Pain Only, or Both

	Overall	None	Problematic substance use only	Chronic pain only	Both	*p*-value*
	*n* (%)	*n* (%)	*n* (%)	*n* (%)	*n* (%)	
	3039 (100.0)	827 (27.2)	352 (11.6)	1129 (37.2)	731 (24.1)	
Accessibility/coordination experience						
Negative	812 (26.72)	144 (17.41)	67 (19.03)	351 (31.09)	250 (34.20)	< 0.001
Cooperation among clinicians experience						
Negative	777 (25.57)	142 (17.17)	61 (17.33)	341 (30.20)	233 (31.87)	< 0.001
Relationship to provider experience						
Negative	817 (26.88)	145 (17.53)	64 (18.18)	361 (31.98)	247 (33.79)	< 0.001
Homeless specific needs experience						
Negative	1,261 (41.49)	264 (31.92)	124 (35.23)	514 (45.53)	359 (49.11)	< 0.001
Overall experience						
Negative	1,036 (34.09)	182 (22.01)	89 (25.28)	446 (39.50)	319 (43.64)	< 0.001

^*^p-values are from *x*^2^

The association of chronic pain and unfavorable care experience remained in multivariable analysis (Table 4). Results of unadjusted analyses are available in Supplemental Table 4. Respondents with chronic pain alone or both pain and problematic substance use were more likely to report unfavorable experiences, compared to patients with neither (aOR = 1.56, 95%CI = 1.34–1.80; and aOR = 1.50, 95% CI = 1.28–1.77, respectively). Respondents with problematic substance use alone did not have increased likelihood of unfavorable experience (aOR = 0.98, 95%CI = 0.81–1.19) compared to respondents with neither.

**Table 4:** Logistic Regression Modeling an Unfavorable PC Experience Among VHE Receiving Primary Care in a Homeless Patient Aligned Care Team (*n* = 3039)

	**Reference**	**aOR**	**95% CI**	***p*-value**
Race	Caucasian			
African American		0.99	0.86–1.12	
Hispanic		0.85	0.71–1.03	0.27
Other		0.88	0.73–1.06	
Marital status	Never married			
Married/partnered		1.09	0.91–1.3	
Divorced/separated		0.92	0.81–1.05	**0.02**
Widowed		1.27	0.99–1.61	
Sex: Female	Male	**0.65**	**0.51–0.84**	**0.002**
Age	65 +			
18–50		**1.55**	**1.26–1.92**	** < 0.001**
51–64		1.18	0.98–1.43	
Employment status	Employed			
Unemployed		1.07	0.93–1.24	**0.03**
Retired		**1.29**	**1.06–1.56**	
Monthly income < $1000: Yes	No	**1.16**	**1.04–1.31**	**0.01**
Difficulty paying for basics: Yes	No	**1.78**	**1.57–2.02**	** < 0.001**
Chronically homeless: Yes	No	1.11	0.98–1.26	0.17
Criminal record: Yes	No	**1.15**	**1.01–1.30**	**0.04**
Jail/prison in last year: Yes	No	0.97	0.79–1.18	0.46
Primary care usage	Bottom tertile			
Top tertile		**0.86**	**0.75–0.98**	**0.03**
Middle tertile		**0.82**	**0.81–0.95**	
ER usage: Top 10%	Bottom 90%	**1.56**	**1.30–1.87**	** < 0.001**
Social support: Low	High	**2.09**	**1.86–2.34**	** < 0.001**
Mental distress: High	Low	**1.54**	**1.36–1.74**	** < 0.001**
Self-reported health: Poor/fair	Good/very good/excellent	**1.40**	**1.25–1.58**	** < 0.001**

*aOR* adjusted odds ratio

### Analyses Restricted to Chronic Pain

Among VHE with chronic pain (*n* = 1860), those with unfavorable primary care experiences tended to be younger, to report low income and difficulty affording basic needs, and to report a criminal justice involvement, higher psychological distress, poor social support, and poor health (Supplemental Table 5). Persons receiving LTOT were less likely to have an unfavorable primary care experience, when compared to those without LTOT (22% versus 42%). Recipients and non-recipients of other therapies did not differ statistically regarding unfavorable experience.

In adjusted models (Table 5), VHE with chronic pain on LTOT were less likely to report unfavorable care experience (OR = 0.49, 95%CI = 0.34–0.69) than VHE not on LTOT. VHE with chronic pain with a visit to occupational therapy were also less likely to report unfavorable care experience (OR = 0.83 95%CI = 0.70–0.98); other pain care services (pain clinic, physical therapy, any active therapy) were not significant.

**Table 5:** Logistic Regression Modeling an Unfavorable PC Experience Among VHE Self-reporting Chronic Pain Including Receipt of Pain-Related Services (*n* = 1860)

	Reference	aOR	95% CI	*p*-value
Race	Caucasian			
African American		0.98	0.83–1.16	
Hispanic		**0.76**	**0.60–0.97**	0.13
Other		0.90	0.72–1.12	
Marital status	Never married			
Married/partnered		1.17	0.95–1.45	
Divorced/separated		0.90	0.77–1.06	**0.01**
Widowed		1.27	0.93–1.72	
Sex: Female	Male	**0.74**	**0.55–0.99**	**0.04**
Age	65 +			
18–50		**1.81**	**1.38–2.37**	** < 0.001**
51–64		**1.45**	**1.14–1.85**	
Employment status	Employed			
Unemployed		1.03	0.85–1.24	**0.001**
Retired		**1.47**	**1.15–1.89**	
Monthly income < $1000: Yes	No	**1.21**	**1.05–1.40**	**0.01**
Difficulty paying for basics: Yes	No	**1.92**	**1.65–2.23**	** < 0.001**
Chronically homeless: Yes	No	**1.19**	**1.01–1.39**	**0.04**
Criminal record: Yes	No	1.09	0.94–1.28	**0.42**
Jail/prison in last year: Yes	No	1.10	0.87–1.40	0.23
PC use	Bottom tertile			
Top tertile		1.01	0.85–1.21	0.85
Middle tertile		1.06	0.87–1.28	
ER usage: Top 10%	Bottom 90%	**1.71**	**1.37–2.14**	** < 0.001**
Social support: Low	High	**1.99**	**1.73–2.31**	** < 0.001**
Mental distress: High	Low	**1.34**	**1.16–1.56**	** < 0.001**
Self-reported health: Poor/fair	Good/very good/excellent	**1.49**	**1.30–1.72**	** < 0.001**
SUD: Yes	No	0.97	0.84–1.12	0.66
LTOT: Yes	No	**0.49**	**0.34–0.69**	** < 0.001**
Pain clinic: Any	None	1.04	0.81–1.35	0.75
Occupational therapy: Any	None	**0.83**	**0.70–0.98**	**0.03**
Physical therapy: Any	None	0.99	0.84–1.16	0.87
Active therapy: Any	None	0.96	0.77–1.20	0.74

*aOR* adjusted odds ratio

### PCQ-H Survey Items with the Highest Unfavorable Care Ratings by Chronic Pain

The greatest magnitude in difference in dissatisfaction ratings between those with and without chronic pain occurred in how a provider “makes decisions based on what will truly help me” (Table 6). Other items included wait times and a concern about lack of communication between health care providers. Two items regarding perceptions of how one’s provider “takes my health concerns seriously” and “never doubts my health needs” were in the top ten items with the greatest difference in rates of dissatisfaction.

**Table 6:** Survey Responses Reflecting Unfavorable Experience When Comparing Persons With and Without Chronic Pain, Ranked by the Magnitude of the Difference

Item	% difference between those with and without chronic pain	*X*^2*^
My primary care provider makes decisions based on what will truly help me	13.9% vs. 4.3%	27
I have to wait too long to get the health care services my primary care provider thinks I need	35.7% vs. 20.6%	26
I have been frustrated by lack of communication among my primary care and other health care providers	33.4% vs. 18.8%	25.1
My primary care provider takes my health concerns seriously	11% vs. 3.2%	24.6
I can get enough of my primary care providers time if I need it	24.5% vs. 12.4%	21.8
It is often difficult to get health care at this place	15.6% vs. 7.3%	19.4
My primary care provider makes sure health care decisions fit with other challenges in my life	15.5% vs. 6.1%	19
My primary care provider never doubts my health needs	17.3% vs. 7.6%	18.1
My primary care and other health care providers need to communicate with each other more	57.3% vs. 45.1%	17.6
At this place, I always have to choose between health care and dealing with other challenges in my life	38.5% vs. 26.8%	16.5

^*^p < 0.001 for all

## DISCUSSION

We found that chronic pain, but not problematic substance use, was associated with a greater likelihood of an unfavorable primary care experience. Among VHE with chronic pain, two types of pain treatments (occupational therapy, long-term opioid therapy) were associated with better primary care ratings. The analysis of individual survey items offers additional insight.

The results highlight opportunities to improve primary care experience for VHE with pain — lessons that might translate to any clinic serving homeless populations. There are conceptual reasons for why chronic pain was associated with unfavorable care experience, while problematic substance use was not. Often, clinicians and patients share a common notion of SUD as diseases that are identifiable and treatable (even if the “brain disease” concept is disputed^42^).

Conversely, evidence on chronic pain is evolving, which can make it difficult for patients and clinicians to agree: some clinicians may present chronic pain as curable if the relevant injury can be neutralized. However, chronic pain is described by some as a “maladaptive recovery response” with peripheral and central drivers.^43^ Clinicians may have difficulty presenting this idea as part of the process of forming a healing relationship. For patients with expectations of cure and health system distrust, these notions may be hard to share in conversation.

Our findings may reflect differences in how pain and SUD care transpire in VA. The VA’s homeless programs often have strong linkages to addiction programs, which may be engaged prior to primary care. In analyses of respondents’ annotations to the survey, many asserted, unprompted, being “clean” or “in recovery.”^20^ VA has adopted the stepped care model that encourages primary care to be more engaged with patients with problematic substance use, thus making them potentially more comfortable treating this population.^44^ For chronic pain, primary care clinicians may feel ill-equipped to respond because they are often the first point of contact.^45–47^ However, patients’ care ratings may be influenced by the quality of their response.^48,49^

In the subgroup analysis where two pain therapies (LTOT and OT) were associated with a lower chance of unfavorable care experience, the association may not be causal. The therapies did pre-date survey administration, but it is not assured that receiving either therapy caused lower dissatisfaction. We speculate that long-term opioids may have been offered by clinicians who had favorable relationships with those patients.^49^ Data hint at the underlying complexity of this relationship: homeless patients often report feeling mistrusted and stigmatized around opioids.^18^ It is possible that opioids conferred actual benefit for long-term pain^50^ and clinicians who prescribe could be affirming they trust the patient. The finding for occupational therapy may suggest a true benefit for such therapies with pain; however, it could also serve as a marker for high levels of engagement in VA’s vocational services, which favors pain recovery.

Finally, the results point toward specific areas of focus for health systems and clinicians. Health systems can directly address wait time and attempt to improve communication shortfalls among team members. Our post hoc survey item exploration raises concerns. Dissatisfaction on making “decisions based on what will truly help me” implicates an absence of trust. Having long waits for necessary services raises a concern about accessibility. The perception that providers do not communicate well is unsurprising because stigma toward this population is likely to frustrate lines of communication.^46,47^ When patients are referred outside primary care for chronic pain, patients might reasonably hope the problem will be “fixed” by the specialist. But successful resolution is uncommon.^51^

The findings suggest ways to improve care experience for VHE, even if guidance regarding caring for chronic pain is unchanged. Expanding the allocated visit time for clinicians to see patients with chronic pain could enhance communication and reduce wait times. Expedited access to pain experts and pharmacists could enhance these efforts.^52^ Strategies to link pain experts and homeless primary care clinicians could be helpful. Partnering with existing programs like Project ECHO or VA Whole Health could plausibly improve pain care within primary care clinics.^53,54^

This study has limitations. A cross-sectional survey cannot prove that chronic pain generated poorer care ratings. Patients’ ratings of care are subjective, but scholars have written that experience ratings do capture how well those services match the ideal of primary care as laid out by the Institute of Medicine.^55^ A 37.3% response rate is higher than that of several other VA surveys, although not ideal.^24^ While the data were inversely weighted for response probability, nonresponse bias is possible. Some respondents did not respond to the primary outcome and covariates (*n* = 355). The findings are limited to VA but may be applicable in other settings. The PCQ-H instrument itself was derived and validated in non-VA and VA populations.^27^ Additionally, we could not measure pain treatments from non-VA health care systems or self-management strategies. Although our study captured a range of pain-related therapies (i.e., active therapy, occupational therapy, pain clinic) based on CPT codes, ICD9/10 codes, and/or VA clinic stop codes, medication therapy was limited to one (long-term opioids) and a larger, more comprehensive study would devise measures for all medications.

## CONCLUSIONS

This analysis explored the association of pain and problematic substance use to patients’ ratings of primary care experience. Whereas problematic substance use alone does not correlate with poor primary care experiences, chronic pain does. Retaining homeless-experienced patients in primary care could prove important to their health, housing retention, and survival. Our findings support renewed commitment to enhancing responses to chronic pain in primary care.

## Supplementary Information

Below is the link to the electronic supplementary material.

Supplementary file1 (DOCX 46 KB)

## Declarations:

### Disclaimer:

Views expressed in this article are those of the authors alone and do not represent positions or views of the United States Department of Veterans Affairs.
