# Supplementary material for: The Association Between Chronic Pain, Substance use, and Primary Care Experience Among Veterans with Ongoing or Recent Homelessness
Source: J Gen Intern Med. 2024 Oct 15;39(16):3172–81. doi: 10.1007/s11606-024-09078-x (PMC11618259; doi:10.1007/s11606-024-09078-x)
Supplement: Supplementary file 1 — Supplementary file1 (DOCX 46 KB) [file 11606_2024_9078_MOESM1_ESM.docx]

SUPPLEMENTARY INFORMATION

Supplementary Tables/Files:

| **Supplemental Table 1.** Sensitivity Analysis showing multivariable-adjusted odds ratio for overall unfavorable primary care experience according to alternative definitions for overall unfavorable primary care experience | | | |
| --- | --- | --- | --- |
|  | Criterion for Overall Unfavorable Experience based on N of 4 scales qualifying as unfavorable* | | |
|  | 1 or more scales | 2 or more scales | 3 or more scales |
|  | *52.2% met this criterion* | *34.1% met this criterion* | *22.1% met this criterion* |
| Neither chronic pain nor SUD | REF | REF | REF |
| Substance use disorder only | 0.83 (0.70-0.98) | 0.98 (0.81-1.19) | 0.97 (0.77-1.23) |
| Chronic pain only | 1.29 (1.13-1.47) | 1.56 (1.34-1.80) | 1.50 (1.27-1.79) |
| Substance use disorder and chronic pain | 1.40 (1.20-1.62) | 1.50 (1.28-1.77) | 1.30 (1.07-1.57) |
| *In the primary paper the criterion for an “overall” unfavorable primary care experience was met when a patient qualified as having an unfavorable primary care experience on two or more of the four available scales from the Primary Care Quality-Homeless 33 instrument. Among respondents 34.1% met the criterion for 2 or more scales. Here we display how the results vary when the criterion for overall unfavorable experience is met | | | |

| **Supplemental Table 2.** Service Codes Used for Pain-Related Services | | |
| --- | --- | --- |
| **Service** | **Service Code Type** | **Code** |
| Physical Therapy | CPT | 95831 |
| Physical Therapy | CPT | 95832 |
| Physical Therapy | CPT | 95833 |
| Physical Therapy | CPT | 95834 |
| Physical Therapy | CPT | 95851 |
| Physical Therapy | CPT | 95852 |
| Physical Therapy | CPT | 96000 |
| Physical Therapy | CPT | 96125 |
| Physical Therapy | CPT | 97001 |
| Physical Therapy | CPT | 97002 |
| Physical Therapy | CPT | 97036 |
| Physical Therapy | CPT | 97110 |
| Physical Therapy | CPT | 97112 |
| Physical Therapy | CPT | 97113 |
| Physical Therapy | CPT | 97116 |
| Physical Therapy | CPT | 97140 |
| Physical Therapy | CPT | 97150 |
| Physical Therapy | CPT | 97530 |
| Physical Therapy | CPT | 97533 |
| Physical Therapy | CPT | 97750 |
| Physical Therapy | CPT | 97799 |
| Physical Therapy | CPT | G0151 |
| Physical Therapy | CPT | G0237 |
| Physical Therapy | CPT | G0238 |
| Physical Therapy | clinic_stop_code | 177 |
| Physical Therapy | clinic_stop_code | 201 |
| Physical Therapy | clinic_stop_code | 205 |
| Active Therapy | CPT | 97005 |
| Active Therapy | CPT | 97006 |
| Active Therapy | CPT | G0176 |
| Active Therapy | CPT | H2032 |
| Active Therapy | CPT | S9449 |
| Active Therapy | CPT | S9451 |
| Active Therapy | CPT | S9970 |
| Active Therapy | ICD9_Procedure | 93.81 |
| Active Therapy | clinic_stop_code | 202 |
| Active Therapy | clinic_stop_code | 214 |
| Active Therapy | clinic_stop_code | 372 |
| Active Therapy | clinic_stop_code | 373 |
| Occupational Therapy | ICD9_Procedure | 93.83 |
| Occupational Therapy | clinic_stop_code | 206 |
| Occupational Therapy | clinic_stop_code | 207 |
| Occupational Therapy | clinic_stop_code | 207 |
| Occupational Therapy | clinic_stop_code | 208 |
| Occupational Therapy | clinic_stop_code | 208 |
| Occupational Therapy | clinic_stop_code | 208 |
| Occupational Therapy | clinic_stop_code | 213 |
| Occupational Therapy | clinic_stop_code | 222 |
| Occupational Therapy | clinic_stop_code | 223 |
| Occupational Therapy | clinic_stop_code | 228 |
| Occupational Therapy | clinic_stop_code | 230 |
| Pain Clinic | clinic_stop_code | 420 |

| **Supplemental Table 3.** Comparison of Veterans included and excluded in study cohort | | | | |
| --- | --- | --- | --- | --- |
|  | Overall | Included in Analytic Sample | Not Included in Analytic Sample | p-value* |
|  | n (%) | n (%) | n (%) |  |
|  | 3,394 (100.0) | 3,039 (89.5) | 355 (10.5) |  |
| Race |  |  |  |  |
| African American | 1,377 (40.57) | 1,233 (40.57) | 144 (10.46) |  |
| Caucasian | 1,312 (38.66) | 1,183 (38.93) | 129 (9.83) | 0.68 |
| Hispanic | 358 (10.55) | 318 (10.46) | 40 (11.17) |  |
| Other | 335 (9.87) | 295 (9.71) | 40 (11.94) |  |
| Marital Status |  |  |  |  |
| Married/Partnered | 488 (14.38) | 446 (14.68) | 42 (8.61) |  |
| Never Married | 972 (28.64) | 855 (28.13) | 117 (12.04) | 0.76 |
| Divorced/Separated | 1,657 (48.82) | 1,501 (49.39) | 156 (9.41) |  |
| Widowed | 215 (6.33) | 189 (6.22) | 26 (12.09) |  |
| Sex |  |  |  |  |
| Female | 185 (5.54) | 164 (5.48) | 21 (11.35) | 0.63 |
| Male | 3,153 (94.46) | 2,830 (94.52) | 323 (10.24) |  |
| Age |  |  |  |  |
| 18–50 | 906 (26.69) | 797 (26.23) | 109 (12.03) | **0.04** |
| 51-64 | 1,923 (56.66) | 1,744 (57.39) | 179 (9.31) |  |
| 65+ | 565 (16.65) | 498 (16.39) | 67 (11.86) |  |
| Education |  |  |  |  |
| HS/GED | 1,406 (42.96) | 1,245 (42.30) | 161 (11.45) | **0.02** |
| More than HS/GED | 1,867 (57.04) | 1,698 (57.70) | 169 (9.05) |  |
| Employment Status |  |  |  |  |
| Employed | 711 (21.58) | 623 (21.00) | 88 (12.38) |  |
| Unemployed | 1,878 (57.00) | 1,717 (57.89) | 161 (8.57) | **0.006** |
| Retired | 706 (21.43) | 626 (21.11) | 80 (11.33) |  |
| Monthly Income < $1,000 |  |  |  |  |
| Yes | 1,522 (46.16) | 1,355 (45.81) | 164 (10.78) | 0.30 |
| No | 1,775 (53.84) | 1,603 (54.19) | 172 (9.69) |  |
| Difficulty Paying for Basics |  |  |  |  |
| Yes | 868 (26.14) | 771 (25.86) | 97 (11.18) | 0.26 |
| No | 2,452 (73.86) | 2,211 (74.14) | 241 (9.83) |  |
| Chronically Homeless |  |  |  |  |
| Yes | 748 (22.04) | 669 (22.01) | 79 (10.56) | 0.92 |
| No | 2,646 (77.96) | 2,370 (77.99) | 276 (10.43) |  |
| Criminal Record |  |  |  |  |
| Yes | 859 (25.78) | 786 (26.08) | 73 (8.50) | 0.23 |
| No | 2,473 (74.22) | 2,228 (73.92) | 245 (9.91) |  |
| Jail/Prison in last year |  |  |  |  |
| Yes | 253 (7.54) | 228 (7.52) | 25 (9.88) | 0.90 |
| No | 3,102 (92.46) | 2,803 (92.48) | 299 (9.64) |  |
| Primary Care Usage |  |  |  |  |
| Top Tertile | 914 (26.93) | 794 (26.13) | 120 (13.13) |  |
| Middle Tertile | 1,184 (34.89) | 1,066 (35.08) | 118 (9.97) | **0.006** |
| Bottom Tertile | 1,296 (38.19) | 1,179 (38.80) | 117 (9.03) |  |
| Emergency Room Usage |  |  |  |  |
| Top 10% | 3,109 (91.60) | 2,777 (91.38) | 332 (10.68) | 0.17 |
| Bottom 90% | 285 (8.40) | 262 (8.62) | 23 (8.07) |  |
| Social Support |  |  |  |  |
| High | 2,019 (60.58) | 1,852 (61.53) | 167 (8.27) | **<.001** |
| Low | 1,314 (39.42) | 1,158 (38.47) | 156 (11.87) |  |
| Mental Distress |  |  |  |  |
| High | 1,113 (33.32) | 1,002 (33.15) | 111 (9.97) | 0.50 |
| Low | 2,227 (66.68) | 2,021 (66.85) | 206 (9.25) |  |
| Self -Reported Health |  |  |  | 0.52 |
| Poor/Fair | 1,508 (46.29) | 1,364 (46.47) | 144 (9.55) |  |
| Good/Very Good/Excellent | 1,750 (53.71) | 1,571 (53.53) | 179 (10.23) |  |
| * p-values are from t-tests or *x*^2^, as appropriate. | | | | |

| **Supplemental Table 4:** Unadjusted associations with unfavorable PC experience among VHE receiving primary care in a Homeless Patient Aligned Care Team (n = 3,039) | | | | |
| --- | --- | --- | --- | --- |
|  | **Reference** | **OR*** | **95% CI** | ***p*-value** |
| Race | Caucasian |  |  |  |
| African American |  | 0.93 | 0.83-1.05 | 0.26 |
| Hispanic |  | 0.96 | 0.81-1.14 |  |
| Other |  | 1.11 | 0.93-1.31 |  |
| Marital Status | Never Married |  |  |  |
| Married/Partnered |  | 0.98 | 0.87-1.10 | 0.82 |
| Divorced/Separated |  | 0.99 | 0.85-1.16 |  |
| Widowed |  | 1.08 | 0.87-1.34 |  |
| Sex: Female | Male | 0.69 | 0.55-0.87 | **0.002** |
| Age | 65+ |  |  |  |
| 18–50 |  | **1.75** | **1.48-2.07** | **<0.001** |
| 51-64 |  | **1.29** | **1.10-1.52** |  |
| Employment Status | Employed |  |  |  |
| Unemployed |  | **1.56** | **1.37-1.77** | **<0.001** |
| Retired |  | **1.22** | **1.03-1.43** |  |
| Monthly Income < $1,000: Yes | No | 1.44 | 1.31-1.59 | **<0.001** |
| Difficulty Paying for Basics: Yes | No | **2.61** | **2.34-2.92** | **<0.001** |
| Chronically Homeless: Yes | No | **1.59** | **1.41-1.78** | **<0.001** |
| Criminal Record: Yes | No | **1.47** | **1.32-1.64** | **<0.001** |
| Jail/Prison in last year: Yes | No | **1.44** | **1.21-1.81** | **<0.001** |
| Primary Care Usage | Bottom Tertile |  |  |  |
| Top Tertile |  | 1.44 | 1.21-1.81 | .250 |
| Middle Tertile |  | 1.44 | 1.21-1.81 |  |
| ER Usage: Top 10% | Bottom 90% | **1.85** | **1.49-2.06** | **<0.001** |
| Social Support: Low | High | **2.89** | **2.60-3.20** | **<0.001** |
| Mental Distress: High | Low | **2.65** | **2.39-2.95** | **<0.001** |
| Self -Reported Health: Poor/Fair | Good/Very Good/Excellent | **2.03** | **1.84-2.25** | **<0.001** |
| Primary Covariates | Neither Chronic Pain or Problematic Substance use |  |  |  |
| Problematic Substance Use Only |  | **1.27** | **1.06-1.53** | **<0.001** |
| Chronic Pain Only |  | **2.18** | **1.91-2.50** |  |
| Both** |  | **2.64** | **2.28-3.05** |  |
| *OR= odds ratio  **Both Problematic Substance Use and Chronic Pain endorsed on survey | | | | |

| **Supplemental Table 5:** Differences in PC Experience in a cohort of Veterans with Chronic Pain | | | | |
| --- | --- | --- | --- | --- |
|  | Overall | Negative PC Experience | Neutral/Positive PC Experience | p-value |
|  | n (%) | n (%) | n (%) |  |
|  | 1,860 (100.0) | 765 (41.1) | 1,095 (58.9) |  |
| Race |  |  |  |  |
| African American | 776 (41.72) | 304 (39.18) | 472 (60.82) |  |
| Caucasian | 679 (36.51) | 286 (42.12) | 393 (57.88) | 0.47 |
| Hispanic | 199 (10.70) | 81 (40.70) | 118 (59.30) |  |
| Other | 197 (10.59) | 88 (44.67) | 109 (55.33) |  |
| Marital Status |  |  |  |  |
| Married/Partnered | 308 (16.56) | 123 (39.94) | 185 (60.06) |  |
| Never Married | 460 (24.73) | 198 (43.04) | 262 (56.96) | 0.70 |
| Divorced/Separated | 944 (50.75) | 377 (39.94) | 567 (60.06) |  |
| Widowed | 116 (6.24) | 49 (42.24) | 67 (57.76) |  |
| Sex |  |  |  |  |
| Female | 117 (6.40) | 40 (34.19) | 77 (65.81) | 0.13 |
| Male | 1,712 (93.60) | 706 (41.24) | 1,006 (58.76) |  |
| Age |  |  |  |  |
| 18–50 | 459 (24.68) | 223 (48.58) | 236 (51.42) |  |
| 51-64 | 1,105 (59.41) | 451 (40.81) | 654 (59.19) | **0.005** |
| 65+ | 269 (14.46) | 91 (33.83) | 178 (66.17) |  |
| Education |  |  |  |  |
| HS/GED | 765 (42.62) | 300 (39.22) | 465 (60.78) | 0.34 |
| More than HS/GED | 1,030 (57.38) | 427 (41.46) | 603 (58.54) |  |
| Employment Status |  |  |  |  |
| Employed | 311 (17.20) | 109 (35.05) | 202 (64.95) |  |
| Unemployed | 1,159 (64.10) | 486 (41.93) | 673 (58.07) | .09 |
| Retired | 338 (18.69) | 140 (41.42) | 198 (58.58) |  |
| Monthly Income < $1,000 |  |  |  |  |
| Yes | 865 (47.84) | 395 (45.66) | 470 (54.34) | **<0.001** |
| No | 943 (52.16) | 344 (36.48) | 599 (63.52) |  |
| Difficulty Paying for Basics |  |  |  |  |
| Yes | 582 (31.91) | 318 (54.64) | 264 (45.36) | **<0.001** |
| No | 1,242 (68.09) | 429 (34.54) | 813 (65.46) |  |
| Chronically Homeless |  |  |  |  |
| Yes | 465 (25.00) | 229 (49.25) | 236 (50.75) | **<0.001** |
| No | 1,395 (75.00) | 536 (38.42) | 859 (61.58) |  |
| Criminal Record |  |  |  |  |
| Yes | 478 (26.54) | 242 (50.63) | 236 (49.37) | **0.007** |
| No | 1,323 (73.46) | 515 (38.93) | 808 (61.07) |  |
| Jail/Prison in last year |  |  |  |  |
| Yes | 160 (8.63) | 82 (51.25) | 78 (48.75) | **0.003** |
| No | 1,693 (91.37) | 680 (40.17) | 1,013 (59.83) |  |
| Primary Care Usage |  |  |  |  |
| Top Tertile | 421 (22.63) | 173 (41.09) | 248 (58.91) |  |
| Middle Tertile | 643 (34.57) | 263 (40.90) | 380 (59.10) | 0.99 |
| Bottom Tertile | 796 (42.80) | 329 (41.33) | 467 (58.67) |  |
| Emergency Room Usage |  |  |  |  |
| Top 10% | 1,672 (89.89) | 665 (39.77) | 1,007 (60.23) | **<.001** |
| Bottom 90% | 188 (10.11) | 100 (53.19) | 88 (46.81) |  |
| Social Support |  |  |  |  |
| High | 1,040 (56.43) | 319 (30.67) | 721 (69.33) | **<0.001** |
| Low | 803 (43.57) | 434 (54.05) | 369 (45.95) |  |
| Mental Distress |  |  |  |  |
| High | 781 (42.19) | 397 (50.83) | 384 (49.17) | **<0.001** |
| Low | 1,070 (57.81) | 365 (34.11) | 705 (65.89) |  |
| Self -Reported Health |  |  |  |  |
| Poor/Fair | 1,058 (58.91) | 491 (46.41) | 567 (53.59) | **<0.001** |
| Good/Very Good/Excellent | 738 (41.09) | 244 (33.06) | 494 (66.94) |  |
| SUD |  |  |  |  |
| Yes | 731 (39.30) | 319 (43.64) | 412 (56.36) | 0.08 |
| No | 1,129 (60.70) | 446 (39.50) | 683 (60.50) |  |
| LTOT |  |  |  |  |
| Yes | 112 (6.02) | 25 (22.32) | 87 (77.68) | **<0.001** |
| No | 1,748 (93.98) | 740 (42.33) | 1,008 (57.67) |  |
| Occupational Therapy |  |  |  |  |
| Any | 547 (29.41) | 220 (40.22) | 327 (59.78) | 0.61 |
| None | 1,313 (70.59) | 545 (41.51) | 768 (58.49) |  |
| Active Therapy |  |  |  |  |
| Any | 241 (12.96) | 113 (46.89) | 128 (53.11) | 0.06 |
| None | 1,619 (87.04) | 652 (40.27) | 967 (59.73) |  |
| Physical Therapy |  |  |  |  |
| Any | 704 (37.85) | 290 (41.19) | 414 (58.81) | 0.97 |
| None | 1,156 (62.15) | 475 (41.09) | 681 (58.91) |  |
| Pain Care Clinic |  |  |  |  |
| Any | 160 (8.60) | 65 (40.63) | 95 (59.38) | 0.89 |
| None | 1,700 (91.40) | 700 (41.18) | 1,000 (58.82) |  |
|  | | | | |
